# Soil and cherry bacterial communities predict flavor on coffee farms

**DOI:** 10.1038/s41598-025-03665-6

**Published:** 2025-06-03

**Authors:** Steve Kutos, Ruth E. Bennett, Danilo Santos, Esteban Botero-Delgadillo, Carly R. Muletz-Wolz

**Affiliations:** 1 Center for Conservation Genomics, Smithsonian’s National Zoo and Conservation Biology Institute, Washington, DC USA; 2 Migratory Bird Center, Smithsonian’s National Zoo and Conservation Biology Institute, Washington, DC USA; 3 SELVA: Research for Conservation in the Neotropics, Bogota, Colombia

**Keywords:** Bacteria, Coffee, Soil ecology, Microbiome, Agroforestry, Agroecology, Microbial ecology

## Abstract

Coffee (*Coffea arabica*) is a popular beverage worldwide with increasing value placed on sensory qualities as specialty markets grow. Plant-associated microbiomes have emerged as a potential but poorly understood driver of coffee flavor that may interact with other environmental and farm management factors. To determine if coffee plant-associated bacteria are linked with flavor, we collected 320 soil and 320 coffee cherry samples from 22 farms in Cundinamarca, Colombia that varied in farm management system (shade vs. sun-grown) and in flavor (presence vs. absence of a specialty flavor profile). We found, using amplicon sequencing, that soil and cherry bacterial communities differed between sun and shade farms and between farms that did and did not produce specialty flavored coffee, with shade + flavor farms notably distinct. Flavor presence was predicted by the abundance of multiple bacterial strains in soils and cherries, but correlations with flavor differed between sun and shade systems. These bacterial taxa correlated with coffee flavor may guide future efforts to optimize farms for specialty quality characteristics. Overall, the observed complex relationships between plant-associated bacteria and coffee flavor will likely require that initiatives seeking to optimize microbiomes for quality outcomes be tailored to individual farm systems and local conditions.

**Supplementary Information:**

The online version contains supplementary material available at 10.1038/s41598-025-03665-6.

## Introduction

Coffee is a high-value agricultural product critical to millions of tropical farmers’ livelihoods^1^. Over the past century, Arabica coffee (*Coffea arabica*) evolved from a pure commodity into a specialty product with differentiation based on quality, including desired sensory profiles and lack of physical and sensory defects^1–3^. High-quality coffees can earn higher prices and stable buyer relationships, which often improve social, economic, and environmental outcomes for coffee-farming communities^1,3,4^ While post-harvesting processes are critical to coffee quality and flavor development, many sensory characteristics depend on where and how the coffee plant grows, including environmental conditions and farm management practices^5–9^. However, one potential driver of flavor has yet to be fully explored in coffee systems: the plant-associated microbiome^10^.

Plant-associated microorganisms, such as bacteria, both impact and are impacted by local environmental conditions and plant characteristics. Beneficial microorganisms can buffer plants against stressors, increase resistance to herbivory, increase water and nutrient uptake efficiency, and suppress pathogens^11–14^. Plant characteristics like phenology and metabolite production^15^ as well as soil characteristics^16^ are also impacted by the community of plant-associated microbes. Notably, these communities show biogeographical variation in taxonomic composition associated with both deterministic factors (e.g., environmental heterogeneity) and stochastic factors (e.g., ecological drift), and microbial community composition typically varies with distance regardless of scale (e.g., regional or local)^17,18^. These factors provide the conditions for coffee microbial *terroir* in which the plant microbiome is driven by local environmental conditions, which then feedback to impact plant performance and crop sensory qualities^19,20^.

Despite accumulating evidence of a relationship between plant-associated microbial communities and agricultural *terroir*^20^, direct evidence is limited for crop systems beyond wine grapes^21,22^. The coffee cherry microbiome may be particularly important to coffee flavor by producing or altering fruit volatiles during plant growth and/or by colonizing the initial fermentation period during post-harvest processing^20^. Yet as far as we are aware, only one study has explored Arabica coffee microbiomes and quality in pre-fermentation samples^23^. Better understanding the relationship between coffee-associated microbes and flavor is key to supporting farm health and to protecting or engineering a microbial community that optimizes quality^24,25^.

While microbiomes may shape *terroir*, coffee microbial communities and coffee quality are both impacted by farm management practices. Across the tropics, Arabica coffee is primarily grown in two management systems: monocultures exposed to direct sunlight (hereafter: sun farms) or agroforestry systems with shade trees that filter sunlight (hereafter: shade farms)^1,26^. The cultivation intensity associated with sun farms can reduce coffee quality indicators including flavor^27–30^. Removing shade trees may impact coffee quality and flavor by altering the aboveground environment (e.g., increased surface temperatures, decreased relative humidity), the belowground environment (e.g., decreased soil moisture, increased erosion), and through increased agrochemical inputs^1,26,27,31–33^. We previously demonstrated that coffee soil microbiomes vary with farm management system in El Salvador, Peru and Colombia^34,35^, and other studies support this inference^36,37^. However, the interactions between farm system, coffee flavor, and coffee microbiomes have yet to be explored.

To address this knowledge gap, we assessed coffee soil and cherry bacterial communities in 22 Colombian coffee farms that varied in production of a high-quality coffee flavor profile (hereafter: flavor) and in management system (sun or shade farms). First, we sought to assess whether coffee soils and cherries have distinct bacterial communities. Microorganisms that inhabit the coffee cherry experience different environmental conditions than soils (e.g., decreased water and nutrient availability, greater UV radiation, and temperature fluctuations)^38,39^. We thus predicted that cherries would have a distinct microbial community composition and reduced species richness compared to the soil communities, as in Veloso et al. (2020). Next, we asked if farms that produced the high-quality flavor had distinct soil physiochemical properties, soil bacterial communities, or cherry bacterial communities compared to farms that did not produce the high-quality flavor, and if farm management system (sun vs. shade) interacted with observable trends. As intensified agriculture systems can lead to alterations of microbial community composition and diversity^34,35,40^, we predicted that sun farms would have distinct soil and cherry bacterial communities. Finally, we sought to identify bacterial taxa correlated with coffee flavor that could be potentially leveraged to improve and better understand quality outcomes on farms.

## Materials and methods

### Site descriptions and experimental design

**Fig. 1:**
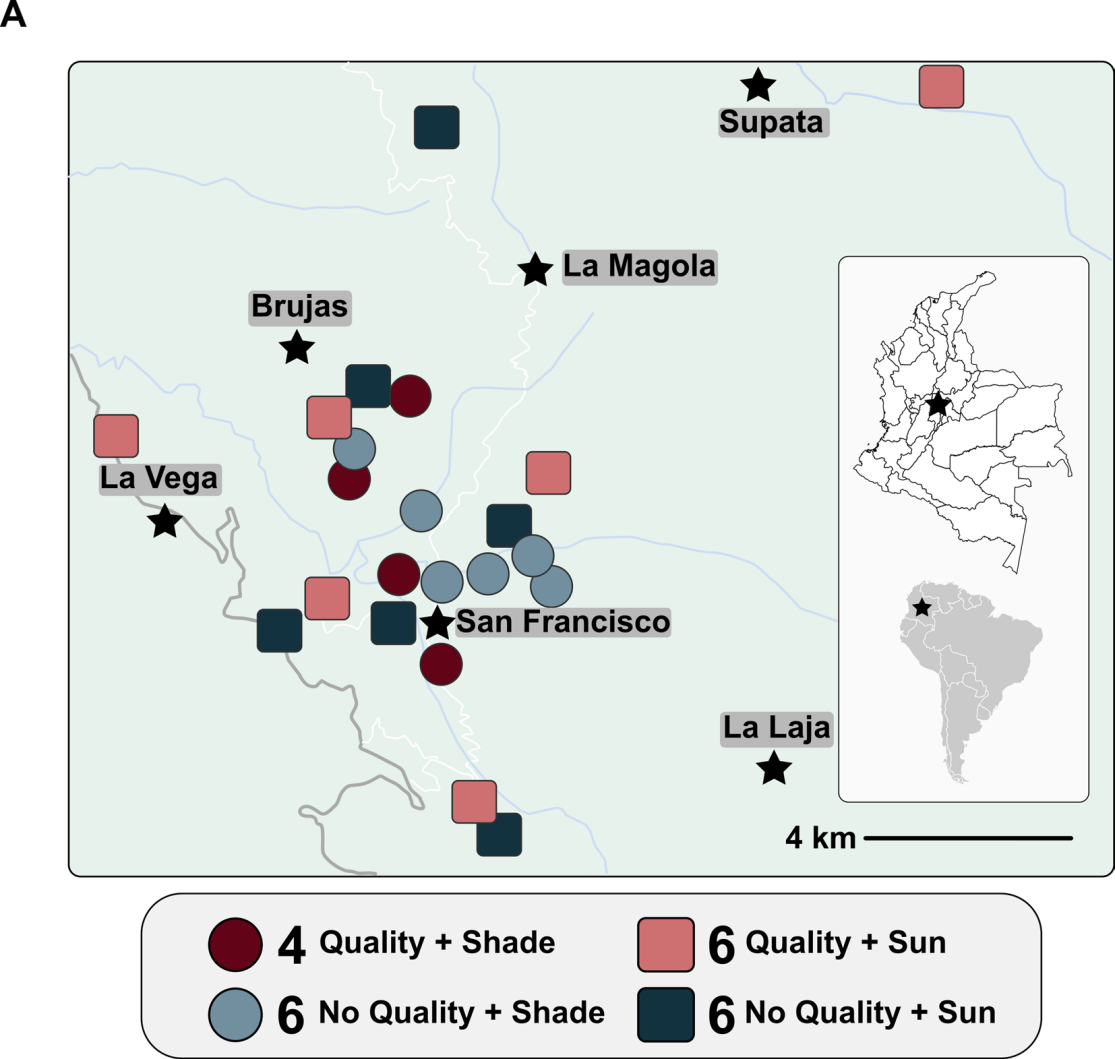
(**A**) Map of study location in the municipality of San Francisco, Cundinamarca, Colombia. Coffee soils and ripe cherries were sampled from shade-grown farms (circles) and sun-grown farms (squares) that differed in production of a flavor profile with specialty market value (“Flavor” - pink/maroon if present, blue if absent). Image created by Steve Kutos with Inkscape (v. 1.3.2 - inkscape.org).

We collected 320 soil and 320 coffee cherry samples from 22 coffee farms located near San Francisco, Cundinamarca, Colombia (4°58’27.3” N, 74°17’21.5” W; Fig. 1). To reduce variation from established microbial distance-decay patterns, we ensured all farms were within 10 km of each other^17,41^. The mean annual temperature for this area is 17 °C, precipitation (mean 330 mm/year) follows a bimodal pattern, and soils are sandy loams classified as Dystric Gleysols^42^. All samples were taken from Arabica coffee plants of the Castillo variety, which is common in Colombia, to reduce potential variation from different coffee varieties^43^. Coffee is harvested locally during two periods: a fly-crop harvest with low, variable production and a principal harvest with high production, with harvest midpoints in October and May, respectively. To capture both harvest periods, we collected samples from 10 farms in October 2021 and 22 farms in May 2022 (the original 10 plus an additional 12 farms).

Farms were selected for inclusion based on whether they produced high-quality coffee, as defined by presence or absence, during the prior two harvests, of a specialty flavor profile characterized by brown-sugar sensory notes^44–47^. Coffee flavor determinations were performed by a local Colombian coffee distributor (Carcafé Ltda., Bogotá, Colombia) trained to detect and purchase coffees with this profile, which occurs in coffee produced on both sun and shade farms in this region (Carcafe, pers. comm.). We stratified farm selection to ensure equal representation of both farm management systems. We classified sun farms if the coffee was cultivated under less than 20% canopy cover and shade farms if the coffee was cultivated underneath greater than 40% canopy cover^48^. On shade farms, most shade trees belonged to species in the genera *Inga*, *Erythrina*, and *Pseudosamanea*. Overall, our experimental design had four subgroups (Fig. 1): (i) flavor + shade coffee (4 farms), (ii) flavor + sun coffee (6 farms), (iii) no flavor + shade coffee (6 farms), and (iv) no flavor + sun coffee (6 farms). To reduce external sources of variation, all farms belonged to the same agricultural extension network, ensuring farmers received similar technical guidance on agrochemical usage, soil management, and harvest procedures.

### Soil and cherry sampling

At each coffee farm, soil samples were collected using a sterilized tool ~ 10–20 cm away from the stem of 10 randomly selected coffee plants at a soil depth of ~ 5–10 cm. At the same randomly selected coffee plant, a ripe cherry was picked with a sterilized gloved hand and placed in a whirl-pak bag (Pleasant Prairie, WI, USA). Both soil and cherry samples were placed on ice, then stored at -20℃ until they were transported with dry ice to the Smithsonian’s Center for Conservation Genomics (Smithsonian’s National Zoo & Conservation Biology Institute, Washington, DC, USA). Collected samples fell under a USDA APHIS permit - P330-22-00099.

### Soil physiochemical characteristics and analyses

At each farm, soils from three points within our sampling area were aggregated together for soil analysis in May 2022. Soil characteristics and nutrients were analyzed for elemental concentrations and physical characteristics via inductively coupled plasma optical emission spectrometry at Soil Health Assessment MegaLab (Yara International, Barranquilla, Colombia). Each soil characteristic was compared among the four treatment subgroups (Fig. 1) with ANOVAs using the *lm* function on log-transformed data with a Bonferroni correction.

### DNA extraction and PCR

Genomic DNA was extracted from 250 mg of soil using the Qiagen DNeasy Powersoil Pro kit (Mississauga, Canada). Cherries were first surface cleaned with a 10% bleach solution and washed with sterile water, then cut in half with a sterile blade then added to a sterilized mortar filled with liquid nitrogen. After 30 s, the half cherry fruit and seed were ground down until powdered. For each sample, ~ 0.3 g of this powder was added to a bead-tube and genomic DNA was also processed using the Powersoil Pro kit. Amplification targeted the 16 S rRNA region using the primers 515 F-Y and 939R^49,50^ under the following conditions: 95 °C for 3 min, followed by 25 cycles of 98 °C for 20 s, 62 °C for 15 s, 72 °C for 15 s, and 72 °C for 1 min. PCRs were completed in duplicate 25 µl reactions containing: 12.5 µl KAPA HiFi HotStart-ReadyMix (Roche, Rotkreuz, Switzerland), 7.5 µl sterile DIH_2_O, 1.0 µl BSA, 1.0 µl 10mM of forward and reverse barcoded primers, and 1 µl extracted DNA on an BioRad T100 Thermocycler (Hercules, CA, USA). In each PCR for cherries, we also included 1 µl of both mPNA (*anti-mitochondrial peptide nucleic acid*) and pPNA (*anti-plastid peptide nucleic acid*) PCR blockers (PNA Bio, Newbury Park, CA, USA) to reduce amplification of mitochondrial and chloroplast DNA^51^. The amplicons were purified using Sera-Mag Speedbeads (GE-Healthcare, Chicago, IL, USA). DNA quantification was completed using a Qubit 4.0 Fluorometer (ThermoFisher, Carlsbad, CA, USA) and an Agilent Technologies 4200 tapestation (Santa Clara, CA, USA). Bacterial library pools were pooled equimolar and then sequenced on two 2 × 300 Illumina MiSeq runs for soils and one 2 × 300 run for cherries at the Smithsonian’s Center for Conservation Genomics.

### Bioinformatics and statistics

Demultiplexed FASTQs were processed using a QIIME2 pipeline (Release: 2023.2)^52^. Sequences were processed through DADA2 to remove poor quality reads and primers^53^. Processed sequences were assigned taxonomic associations using the SILVA database (Version 138)^54^. Any ASV not classified as bacteria at the kingdom level were removed from analyses. Soil sequences were rarified to a depth of 10,125 per sample and cherries were rarified to a depth of 4599 sequences per sample due to the large imbalance in sequence depth across samples. To reduce potential contaminant ASVs we used *decontam* during the filtering process^55^. Overall, 58 soil samples and 30 cherry samples were lost because of low sequencing coverage after all filtering.

All statistics were completed in R (Version 4.3.2) primarily using the vegan and phyloseq packages^56,57^. We compared soil and cherry bacterial community structure between the two harvest periods and found significant differences in community composition (*p* < 0.05). Therefore, we analyzed each harvest period separately. We compared alpha diversity metrics (Shannon diversity index and ASV observed richness) using linear mixed effects models (lmer in the *lme4* R package) with farm management (sun vs. shade) and quality flavor (presence vs. absence) and their interaction as explanatory variables and with farm ID as a random effect to account for pseudo-replication. Beta diversity was measured using PERMANOVAs (adonis2 in the *vegan* package) between samples on log-transformed ASV counts using Bray-Curtis dissimilarity with farm ID as the random effect. NMDS ordinations were used for visualizations. Homogeneity of dispersions was checked prior to running the PERMANOVAs. Post hoc pairwise comparisons between differences in the PERMANOVA were completed using the *pairwiseAdonis* package^58^. Soil characteristics were correlated with the soil bacterial community using redundancy analysis as well as partial Mantel tests via the vegan package with the third matrix being Haversine distances based on geolocation.

Bacterial differential abundance analysis was focused on flavor presence in soil and cherry principal harvest farm samples using Analysis of Compositions of Microbiomes with Bias Correction (ANCOM-BC2) with a Holm correction for multiple testing and farm ID as the random effect^59^. We present results here for the principal harvest, as it has greater economic significance. Only ASVs that were found in 25% of samples and more than 200 reads within each subgroup were included (soil + shade: *n* = 439 ASVs, soil + sun: *n* = 543 ASVs, cherry + shade: *n* = 138 ASVs, cherry + sun: *n* = 145 ASVs). In flavor comparisons for cherry bacterial communities, we were particularly interested in lactic acid bacterial families (Listeriaceae, Enterococcaceae, Lactobacillaceae) as they can modulate pH, produce metabolites suggested to improve coffee flavor, and can alter the growth of yeasts during fermentation^60–62^, and acetic acid bacteria (Acetobacteraceae, Enterobacteriaceae) as they can produce metabolites during the fermentation process that can decrease coffee flavor quality^63,64^. Visualization of the ANCOM results were displayed as *log-fold change* of bacterial ASVs or genera across the four subgroups. For bacterial genera visualization, the number of bacterial ASVs within a genus are indicated for those ASVs that were statistically significant between flavor/no flavor comparisons within that genus (Fig. S1). To explore ASVs shared between soil and cherries in farms with the quality flavor, a bipartite network among the top 100 most abundant ASVs was created using the *bipartite* r package. We used *ggplot2* and *igraph* packages to visualize each network using the Davidson-Harel layout.

## Results

### Soil characteristic differences across farms

No soil properties differed significantly among the four flavor-farm treatment subgroups (*p* > 0.05), although subgroup-specific patterns were present (Table 1). For instance, sun farms regardless of flavor presence tended to have higher microbial biomass, soil respiration, and mineralizable nitrogen, whereas shade + flavor farms tended to have higher potassium and phosphorus (Table 1; *p* > 0.05). No significant correlations were found between soil characteristics and bacterial community composition for any farm system or flavor subset (*p* > 0.05).

**Table 1:** Average of measured soil characteristics within the farms across our four subgroups. Standard deviation is within parentheses.

	Soil characteristics			
	Shade + quality farms	Shade + no quality farms	Sun + quality farms	Sun + no quality farms
Ca (ppm)	1026 ± (1053)	640 ± (399)	552 ± (276)	916 ± (369)
Mg (ppm)	134 ± (144)	70 ± (34.3)	52 ± (23.9)	99 ± (59.4)
B (ppm)	1 ± (0.1)	0.8 ± (0.2)	0.7 ± (0.1)	0.7 ± (0.2)
Fe (ppm)	550 ± (547)	239 ± (197)	392 ± (386)	440 ± (324)
K (ppm)	180 ± (121)	82 ± (55)	67 ± (31)	73 ± (40)
Zn (ppm)	6 ± (4)	16 ± (11)	6 ± (3)	10 ± (5)
P (ppm)	40 ± (40)	10 ± (5)	13 ± (8)	8 ± (3)
Min N (kg N/ha)	34 ± (15)	32 ± (17)	49 ± (15)	49 ± (31)
Microbial Biomass (mg/kg)	1389 ± (680)	1218 ± (511)	1962 ± (722)	1904 ± (1150)
Organic Matter (%)	12 ± (3)	11 ± (5)	10 ± (4)	10 ± (4)
C.E.C. (meq/100 g)	10 ± (7)	8 ± (3)	7 ± (2)	11 ± (4)
Solvita Index Score	42 ± (12)	37 ± (14)	50 ± (11)	47 ± (17)
Soil Respiration (mg/kg)	62 ± (31)	54 ± (23)	88 ± (33)	85 ± (52)
C: N Ratio	12 ± (1)	12 ± (2)	12 ± (1)	12 ± (1)
pH	5 ± (0.4)	5 ± (0.2)	5 ± (0.2)	5 ± (0.4)
Silt (%)	31	30	27	26
Clay (%)	11	13	9	8
Sand (%)	58	56	64	66
Base saturation (%)	47 ± (5)	40 ± (11)	37 ± (11)	47 ± (5)

### Bacterial compositions in soils and cherries

After all filtering steps, we retained 252 soil samples with 31,952 unique bacterial ASVs (2.45 × 10^6^ sequences) and 280 cherry samples with 8,902 ASVs (1.3 × 10^6^ sequences). In soils, taxa that had the highest relative abundances were in the phyla Proteobacteria (mean relative abundance: 29.0%, *n* = 7653 unique ASVs), Actinobacteriota (28.5%, *n* = 5096 ASVs), and Acidobacteriota (9.5%, *n* = 3303 ASVs). In the cherry samples, taxa that had the highest relative abundances belonged to phyla Proteobacteria (74.2%, *n* = 3612 unique ASVs), Actinobacteriota (17.5%, *n* = 1558 unique ASVs), and Bacteroidota (3.4%, *n* = 1895 unique ASVs).

### Bacterial community composition differences between coffee soils and cherries

**Fig. 2:**
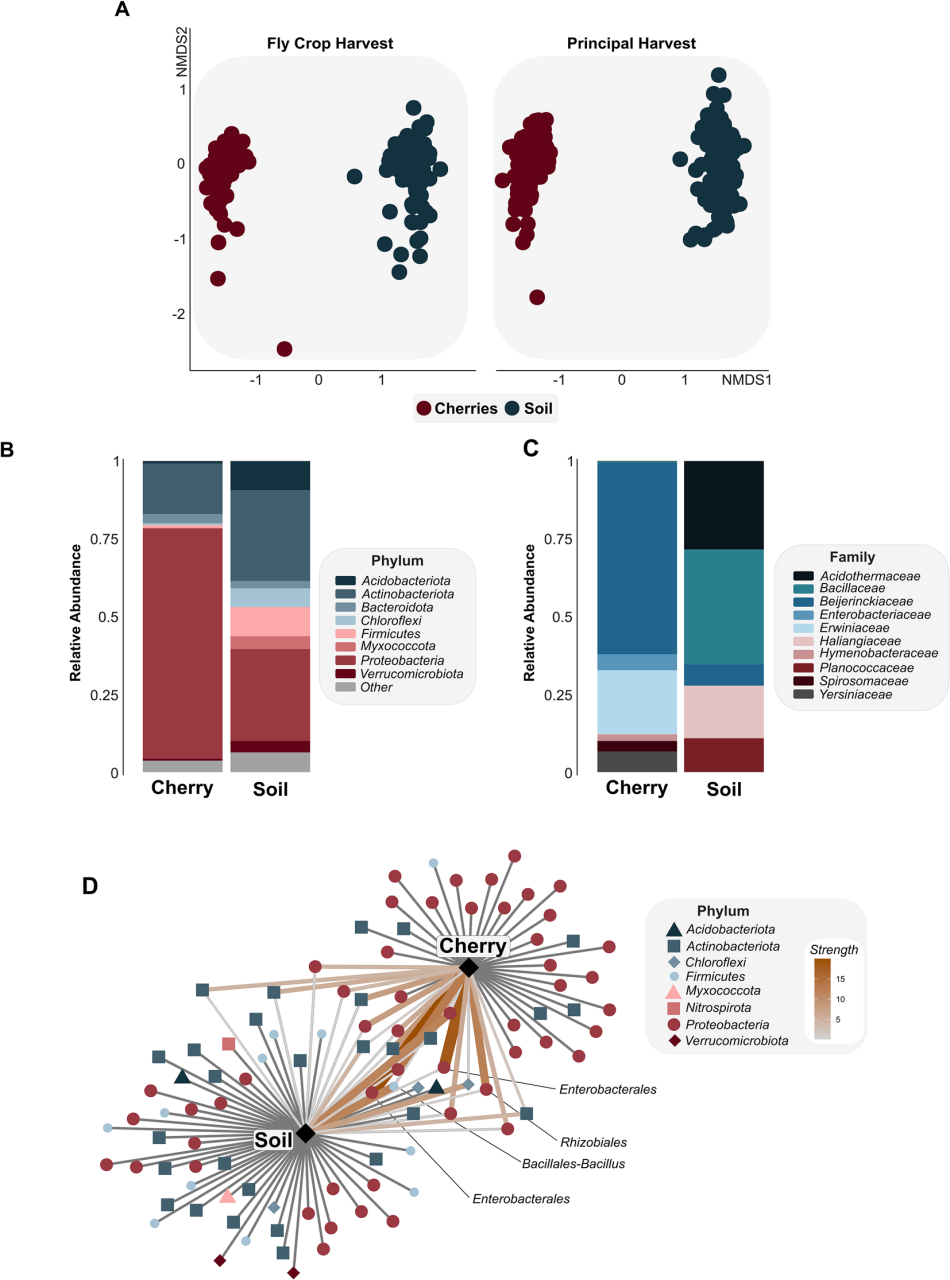
Bacterial diversity between coffee cherries and soils. (**A**) Beta diversity of coffee cherry and soil bacterial communities in two harvest periods. Relative abundance of (**B**) bacterial phyla and (**C**) bacterial families for cherry and soil bacterial communities. (**D**) Bipartite network of the top 100 most abundant bacterial ASVs from farms that produce coffee with a specialty flavor profile.

**Fig. 3:**
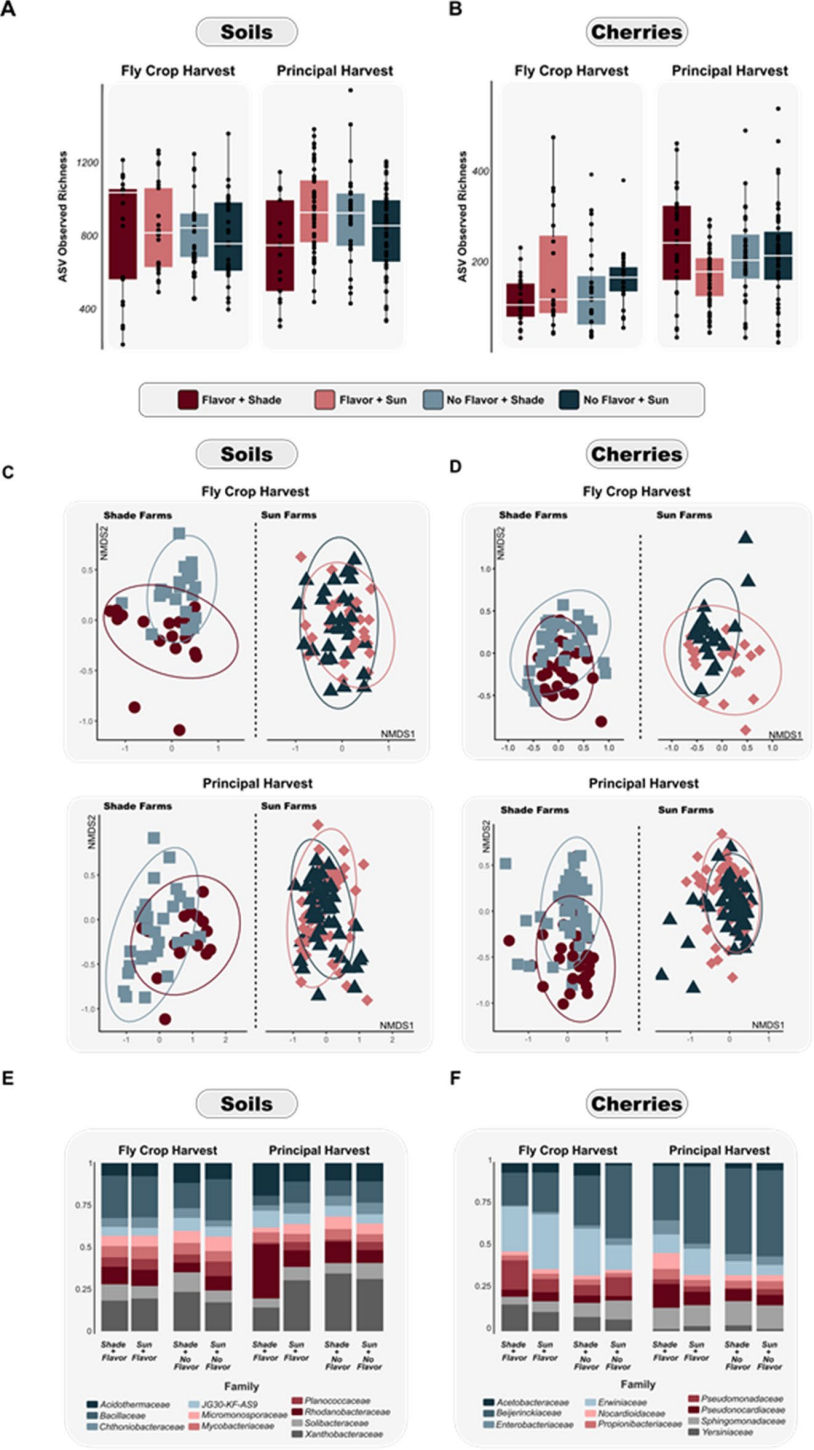
Richness and diversity of coffee cherries and soils among experimental subgroups. (**A**) Soil ASV and (**B**) cherry ASV observed richness for four coffee flavor-farm system subgroups across two harvest periods. (**C**) Beta diversity of soil bacterial communities and (**D**) cherry bacterial communities during two harvest periods for four coffee-flavor-farm system subgroups. Results for each farm management system (sun or shade coffee) presented in separate subplots – full beta diversity plots are shown in Supplemental Fig. 2. (**E**) Relative abundance of bacterial families from soils and (**F**) coffee cherries for each of four coffee flavor-farm system subgroups by harvest period.

Across all farms, soil and cherry samples only shared 3% of ASVs, although they shared 40% of bacterial families. Bacterial community composition between soils and cherries was significantly different (*Bray–Curtis dissimilarity*: *F* = 116.3, *p* < 0.001, r^2^ = 0.17; Fig. 2a; Supplementary Fig. S1). Soils had on average 850 ASVs per sample (+/- 250 SD), whereas cherries had on average 180 ASVs per sample (+/- 100 SD), which differed in Shannon’s diversity (*F* = 758.4, *p* < 0.001) and observed ASV Richness (*F* = 1728, *p* < 0.001; Fig. 3a, b). Relative abundances of bacteria at the phylum level differed substantially between coffee soils and cherries (Fig. 2b). For instance, five bacterial phyla tended to have higher relative abundance within coffee soils, including Chloroflexi (11.8X), Verrucomicrobiota (5.4X), Myxococcota (15.7X), Planctomycetota (6.2X), and Acidobacteriota (11.3X; Fig. 2b). Conversely, taxa in the bacterial phylum Proteobacteria were higher in relative abundance on the cherries (Fig. 2b). Some bacterial families that were abundant in either the soil or the cherries were rare or absent in the other sample type. For instance, taxa in the bacterial families Acidothermaceae, Caryophanaceae, Micrococcaceae, Haliangiaceae, and Bacillaceae were found in relatively high abundances in soils but were exceedingly rare in cherry samples (< 0.01% sequences; Fig. 2c). Oppositely, taxa in the families Erwiniaceae, Enterobacteriaceae, Spirosomaceae, Yersiniaceae, and Hymenobacteraceae were found in relatively high abundances on the cherries but were rare in soils (< 0.01% sequences; Fig. 2c). Finally, among the top 100 most abundant ASVs, only 20 were shared among cherries and soils (Fig. 2d), which includes ASVs in the genera *Bacillus*, JG30-KF-AS9, *Nakamurella*, *Friedmanniella*, *Pseudomonas*, *Actinomycetospora*, *Bradyrhizobium*, and two unidentified Gammaproteobacteria ASVs.

### Variation in coffee soil and cherry bacterial community composition among farm system and coffee flavor categories

Neither soil nor cherry bacterial communities in either harvest period differed in Shannon diversity (*p* > 0.05; Fig. 3a, b; Table 2) or ASV observed richness (*p* > 0.05; Fig. 3a, b; Table 2). Soil bacterial community composition, however, was significantly different among the four flavor-farm system subgroups during the fly-crop harvest (*Bray-Curtis - flavor x management*: *F*_*(1,94)*_ = 2.7, *p* < 0.001, r^2^ = 0.03; Fig. 3c; Supplementary Fig. S3; Table 2) and the principal harvest (*Bray-Curtis - flavor x management*: *F*_*(1,153)*_ = 2.9, *p* < 0.001, r^2^ = 0.02; Fig. 3c; Supplementary Fig. S3; Table 2). Sun farm samples within both seasons were not significantly different from each other via the pairwise comparison. Cherry bacterial community composition differed among the four flavor-farm system subgroups during the principal harvest (*Bray-Curtis - flavor x management*: *F*_*(1,183)*_ = 2.8, *p* = 0.002, r^2^ = 0.02; Fig. 3d; Supplementary Fig. S3; Table 2) but not during the fly crop harvest (*Bray-Curtis - flavor x management*: *p* > 0.05; Fig. 3d; Supplementary Fig. S3; Table 2). During the principal harvest, the bacterial community differentiation between flavor and no-flavor farms was greater in shade-grown systems than sun-grown systems (Fig. 3c, d, e, f). Shade-grown, flavor-producing farms also had the most unique bacterial communities of all four subgroups (Fig. 3e, f; Supplementary Fig. S2).

**Table 2:** Bacterial diversity. PERMANOVA results on Bray-Curtis distances and alpha diversity results for soils and coffee cherries within both harvest periods.

	Beta diversity Bray-Curtis distance					Alpha diversity Shannon’s diversity		Alpha diversity Observed ASV richness	
	df	Sum of squares	F	R^2^	p-value	df	p-value	df	p-value
Soil bacterial diversity									
Principal harvest samples									
Presence of flavor	1.153	0.70	2.02	0.01	0.006*	16.135	0.079	16.135	0.18
Management system	1.153	1.54	4.51	0.03	0.001*	16.135	0.62	16.135	0.37
Presence of flavor x management system	1.153	1.00	2.93	0.02	0.001*	16.135	0.08	16.135	0.09
Fly crop harvest samples									
Presence of flavor	1.97	0.86	2.65	0.03	0.001*	8.86	0.55	8.86	0.91
Management system	1.97	0.56	1.72	0.02	0.010*	8.86	0.53	8.86	0.84
Presence of flavor x management system	1.97	0.88	2.72	0.03	0.001*	8.86	0.47	8.86	0.78
Cherry bacterial diversity									
Principal harvest samples									
Presence of quality flavor	1.186	1.30	3.70	0.02	0.001*	6.83	0.42	6.83	0.64
Management system	1.186	1.40	4.10	0.02	0.001*	6.83	0.36	6.83	0.72
Presence of quality flavor x management system	1.186	1.00	2.90	0.02	0.001*	6.83	0.97	6.83	0.68
Fly crop harvest samples									
Presence of quality flavor	1.92	0.80	1.99	0.02	0.02*	16.169	0.69	16.169	0.56
Management system	1.92	0.61	1.62	0.02	0.04*	16.169	0.86	16.169	0.94
Presence of quality flavor x management system	1.92	0.55	1.46	0.02	0.09	16.169	0.9	16.169	0.18

Star denotes significance.

### Relationship between soil bacterial taxa, coffee flavor, and coffee management

We assessed whether the production of high-quality flavored coffee was related to differences in bacterial relative abundances (Fig. 3e). At the family level, we observed bacteria that were differentially abundant in the soils of flavor-producing farms (Fig. 3e). For instance, taxa in the family *S0134* were 3X higher in relative abundance in flavor-producing sun farms during the principal harvest. Also, taxa in the families Sphingomonadaceae and Intrasporangiaceae were both 2X higher in relative abundance in flavor-producing farms regardless of management practice during the fly crop harvest. In the principal harvest, taxa in the families Xanthobacteraceae (3X), JG30-KF-CM45 (2.2X), Bacillaceae (5X), Solirubrobacteraceae (4X), and Caryophanaceae (12X) were all higher in relative abundance in sun farms regardless of flavor presence.

**Fig. 4:**
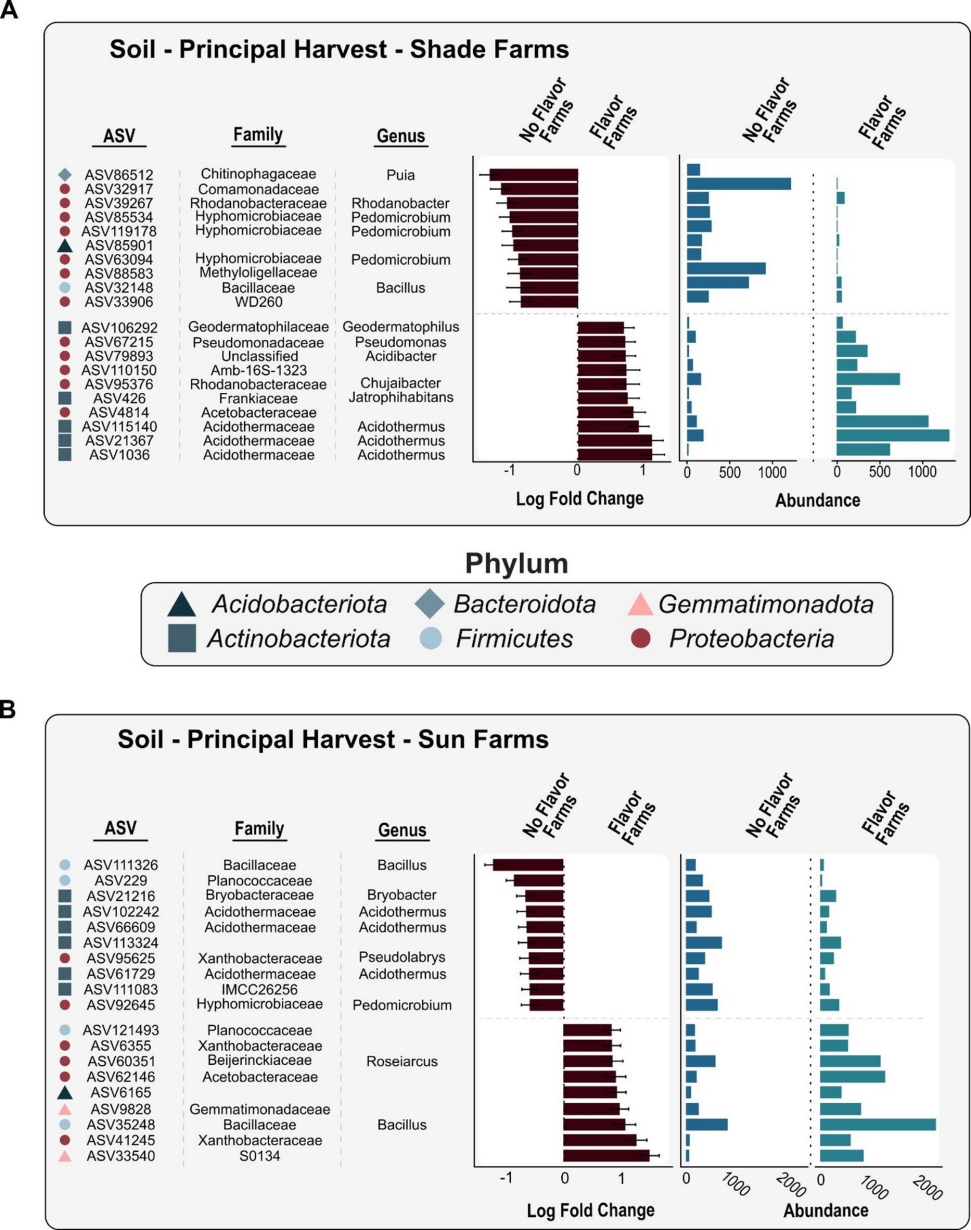
Differential abundance and log fold change of soil bacteria ASVs that significantly associated with the presence or absence of coffee flavor via the ANCOMBC analysis. (**A**) Shade grown coffee farms and (**B**) sun grown coffee farms during the principal harvest period.

Of the 704 soil ASVs evaluated within the principal harvest, 39 were significantly associated with flavor via ANCOMBC (Fig. 4a). For soils from shade farms, ASVs within the genera *Acidothermus*, *Jatrophihabitans*, *Chujaibacter*, *Acidibacter*, *Geodermatophilus*, and *Pseudomonas* were higher in relative abundance on farms that produce the flavor (*adj P* < 0.05; Fig. 4a). Conversely, ASVs in the genera *Puia*, Comamonadaceae (unknown genus), *Rhodanobacter*, Methyloligellaceae (unknown genus), and *Bacillus* were lower in relative abundance if the flavor was not present (*adj P* < 0.05; Fig. 4a). Interestingly, three ASVs in the genera *Acidothermus* were higher in relative abundance in sun farms without flavor; an opposite pattern than what was observed for another *Acidothermus* ASV in shade farms (*adj P* < 0.05; Fig. 4b). Four ASVs in the genus *Pedomicrobium* were higher in relative abundance in soils without flavor in both sun and shade farms (*adj P* < 0.05; Fig. 4b). Finally, we observed zero soil ASVs that were significantly correlated with flavor presence or absence across both harvest periods (*only comparing the 10 farms sampled in both periods*).

### Relationship between cherry bacterial taxa, coffee flavor, and coffee management

Matching soil patterns, we detected distinct patterns in differential abundances of bacteria in the cherries (Fig. 3f). For instance, in the principal harvest, taxa in the family Frankiaceae were 2X more abundant on cherries from flavor-producing farms regardless of management system. Also in the principal harvest, taxa in the families Mycobacteriaceae (2.6X), Labraceae (9X), Sphingomonadaceae (2.2X), Hymenobacteraceae (3X), Chthoniobacteraceae (5X), and Beijerinckiaceae (3.2X) were lower in relative abundance from flavor-producing shade farms compared with the other farm management system-flavor groups (Fig. 3f). We observed no difference in the relative abundance of lactic acid taxa. However, we did observe differences in the relative abundance of acetic acid taxa. Specifically, taxa in the family Acetobacteraceae were 1.8X more abundant on coffee cherries in farms without flavor, regardless of management practice or harvest period (Fig. 3f). Finally, taxa in the family Enterobacteriaceae were 3X more abundant on cherries from farms without flavor, regardless of management practice, but only during the fly crop harvest (Fig. 3f).

**Fig. 5:**
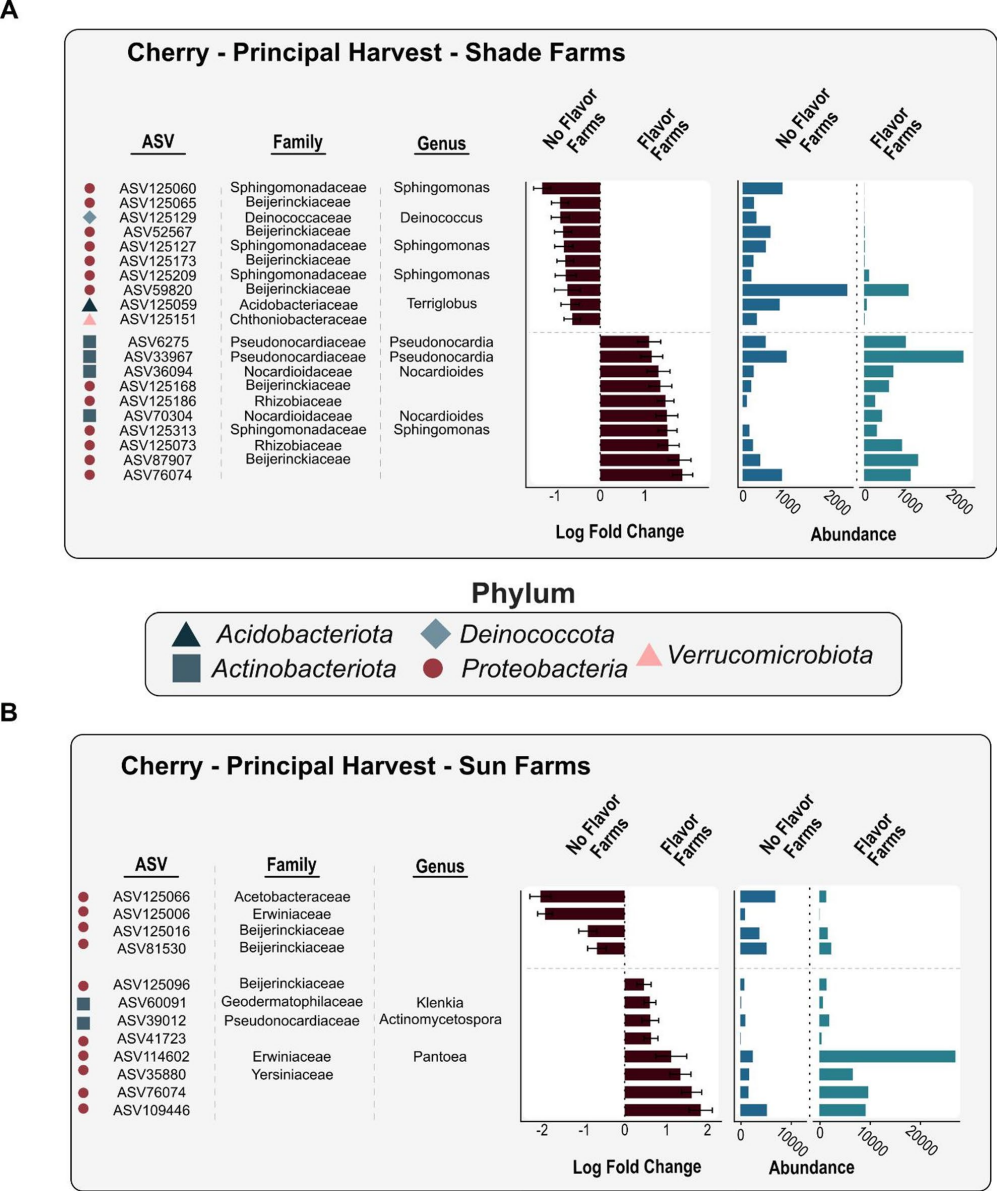
Differential abundance and log fold change of cherry bacteria ASVs that are significantly associated with the presence or absence of coffee flavor via the ANCOMBC analysis. (**A**) Shade grown coffee farms and (**B**) sun grown coffee farms during the principal harvest period.

Of the 137 cherry ASVs evaluated within the principal harvest, 32 were significantly associated with flavor (Fig. 5a). Specifically, two ASVs in the genus *Nocardioides* and two ASVs in the genus *Pseudocardia* had higher relative abundances on cherries from flavor-producing shade farms (*adj P* < 0.05; Fig. 5a). Oppositely, three ASVs in the genus *Sphingomonas* had higher relative abundance in shade farms without flavor (*adj P* < 0.05; Fig. 5a). However, a different ASV in the *Sphingomonas* genus was associated with flavor (*adj P* < 0.05; Fig. 5a). Cherries from sun farms had fewer ASVs that predicted flavor than in shade farms, which included ASVs in the genera *Klenkia*, *Actiomycetospora*, and *Pantoea* (*adj P* < 0.05; Fig. 5b). We observed no overlap in ASVs found in soils and the cherries that were associated with flavor presence. Finally, matching the pattern found in soils, we found zero cherry ASVs that were significantly correlated with flavor presence or absence across both harvest periods.

## Discussion

We explored the relationship between bacterial community composition, flavor presence, and cultivation management of Arabica coffee in Colombia. We showed that coffee farms known to produce a high-quality flavor profile had unique soil and cherry bacterial communities. To our knowledge, this is the first study to demonstrate variation in bacterial communities related to flavor within a single variety of Arabica coffee plants. Our finding that coffee soil and cherry bacterial communities vary with farm management supports previous studies^34,35^, but our results are novel in the finding that the impact of management on bacterial communities can differ between farms that did and did not produce a high-quality flavor profile. We found multiple bacterial markers of flavor within sun or shade systems, particularly taxa in the genera *Acidothermus*, *Chujaibacter*, and *Sphingomonas*. Microbial taxa can affect plant or soil functionality^65,66^, which may in turn lead to variation in quality and flavor. However, the functions of some bacterial taxa described here are unknown or poorly described.

We wish to highlight the bacterial markers of flavor in shade-grown coffee because these farming systems have declined globally due to intensification pressure^27^. Our findings demonstrate that shade-grown, flavor producing coffee farms have unique bacterial communities in this region. As shade farms support ecological functions and biodiversity important to production and human communities^1,26,33,67^, finding ways to increase coffee quality in these systems could improve their economic sustainability and lead to improved social and environmental outcomes. Further, if synthetic bacterial biofertilizers could be developed to increase the likelihood of high-quality outputs in shaded systems, farmers may be more likely to maintain or transition back to shaded farming practices.

Development of these applied microbial biofertilizers in coffee systems requires multiple steps, including experimentation on the ecology and biology of these organisms (e.g. storage viability, inoculation potential, application rate, inoculation longevity), and field trials to assess impact of inoculation on soil microbial composition, plant health, and coffee flavor. Our results provide preliminary guidance on the selection of microbial taxa for future research in this direction. Our results also suggest that biofertilizer impact may depend on farm context. Biofertilizers would likely achieve the greatest impact if they seek to augment abundance of correlated microbial species to the type of farm system where those species serve as key functional taxa. The fact that all taxonomic signatures of flavor identified in this study were specific to farm system (sun or shade) suggests that environmental conditions may inhibit successful inoculation of microbial taxa on farm types with different environmental conditions that the farms where these taxa were identified. Further experiments could confirm whether and how flavor-associated bacterial taxa influence coffee quality in this Colombian region.

We observed surprisingly low variation in soil properties across farms, given evidence that farm intensification alters the soil environment^33,67^. While not significantly different, flavor-producing shade farms had higher concentrations of potassium and phosphorus. More data is needed to confirm this pattern, but these elements promote plant growth as well as fruit development. We also observed that sun farms, regardless of flavor presence, had higher microbial biomass, soil respiration, and mineralizable nitrogen. This might be due to certain microbial taxa proliferating in these soils leading to an increase in biomass and potentially changing the soil functional profile, but this would need further experimentation to confirm. Finally, disagreeing with our expectations and patterns found in other studies^37^, we observed no significant associations between observed soil characteristics and bacterial community composition, which may be due to our low soil sample size per farm and/or the lack of significant difference among our soil variables. Overall, more evidence is needed across spatial distances and time in coffee systems in this and other regions to further explore microbiomes and their relationship to soil properties.

In many agricultural systems, microbiome compositions between the soil and plant-associated tissues are usually distinct^68^, including previous evidence in a coffee system^40^. Our results support this pattern, showing that bacterial communities were strikingly different between coffee cherries and soils, with lower ASV richness in cherries. Interestingly, a small subset of bacterial ASVs did inhabit both the cherry and soil, highlighting an interconnected system in which the soil bacterial community may be an inoculating source for cherry bacterial community in coffee landscapes. However, it is likely that the cherry bacterial community is assembling independently from the soil during fruit development and maturation, with the host plant resident bacterial community potentially being a main inoculation source^69,70^. Bacterial species introductions may also come from pollinator arrivals, precipitation, or humans, and community assembly is likely influenced by priority effects and microbe-microbe interactions, which ultimately leads to unique bacterial community compositions across this landscape^69,70^. This may have important implications for the coffee quality and flavor, as these taxa can be initial colonizers during post-harvesting processes and can combat microbial pathogens during fruit maturation^71–73^. Overall, these results suggest that coffee soils and cherries are colonized and inhabited by varied bacterial taxa through diverse factors leading to distinct community compositions.

We found evidence for coffee microbial *terroir* as soil and cherry bacterial abundances and compositions, in some instances, differed between farms with flavor compared to those without the flavor profile. Studies have shown a relationship between microbial communities and quality in wine and other systems^19,74^, but our results are the first to show this in coffee systems using plants of the same variety. Further, particular bacterial taxa were differentially abundant in soils and cherries from farms with flavor presence and might be correlated with this flavor profile in this region. These findings provide the groundwork for experimentation to determine if these patterns are consistent temporally and spatially and if and how correlated bacterial groups impact coffee plant development, productivity, and coffee flavor.

We identified bacteria with associations with flavor based on their varied relative abundances. For instance, ASVs in the genus *Acidothermus* were associated with flavor presence in shade farms. However, other *Acidothermus* ASVs were associated with flavor absence in sun farms, further bolstering the importance of strain level variation in these communities. Interestingly, a recent study showed that *Acidothermus* species were also associated with the quality of pear fruits^75^. Another group that was correlated with flavor presence, but only in the principal harvest from shade farms, were ASVs in the genus *Chujaibacter*. Not much is known about the functional potential of this group, which was first isolated in 2015^76^. However, a new study showed that the increased abundance of *Chujaibacter* taxa was linked with root metabolite activities vital for blueberry fruit production^77^, suggesting a potential key functional role.

We also identified bacterial taxa in coffee cherries that were correlated with flavor in this system. For instance, taxa in the genera *Pantoea* were more abundant in flavor-producing farms. Curiously, in a 2015 study, bacterial isolates from a species within this genus (*Pantoea coffeiphila*) were correlated with decreased quality and flavor in Arabica coffee^78^. There were also three ASVs within the genus *Sphingomonas* that were more abundant in shade farms without flavor presence. While taxa in this group vary widely in their functions, some species can produce phytohormones, which can influence fruit growth patterns and development^79^. Overall, species and strains within these groups and others associated with flavor are a potential direction for future study in coffee systems to understand their possible functional relation to quality and flavor.

Many bacterial species may be important for the modulation of coffee quality and flavor in post-harvesting processes, which include the lactic acid group which is known to improve flavor^60–62^ and the acetic acid and Enterobacteriaceae groups which are known to decrease flavor^59,64,72,73^. We observed no significant difference in abundance patterns of lactic acid bacteria among our treatments suggesting these taxa can subsist in varied levels of environmental shifts across this region and across farming systems (sun vs. shade grown). However, acetic acid taxa (Acetobacteraceae) and Enterobacteriaceae taxa were more abundant in non-flavor producing farms (Enterobacteriaceae taxa only during the fly crop harvest) suggesting these taxa might be correlated with cherry flavor development. It is possible these taxa can subsist to the fermentation step leading to or assisting in the decrease in coffee flavor; however, this would need to be evaluated. To the best of our knowledge, this is the first study to explore these bacterial groups in a sample comparison of flavor presence and flavor absence in pre-fermentation coffee cherries providing a baseline for future studies.

Shade grown coffee provides many ecological benefits to coffee landscapes as compared to more open sun systems. Recent studies have shown that cultivation intensity does influence coffee soil microbial communities^34,35^. Our results build upon and add to these studies by showing that cultivation method does influence both soil and cherry bacterial communities. In soils, a few genera had higher relative abundances associated with sun farms in the principal harvest including *Bradyrhizobium*,* Pseudolabrys*, *JG30-KF-CM45*, *Bacillus*, *Conexibacter*, *Lysinibacillus*, and *Sporosarcina*. Taxa in these genera are common soil residents that perform key functions including nitrogen fixation (*Pseudolabrys*, *Bradyrhizobium*,* Lysinibacillus*, *Sporosarcina*), carbon substrate degradation (*Conexibacter*), and can promote phytohormone production (*Lysinibacillus*)^80–85^. While further study is needed, the increased relative abundances of taxa in these groups in sun farm soils can potentially affect soil nutrient cycling as well as affect coffee plant development.

Cherry bacterial communities overall had fewer taxa abundance differences between sun and shade farms as compared to soils suggesting these communities are not as influenced by the altered environmental factors seen in intensified coffee systems as compared to the soils. Further, the observed differences in bacterial community compositions were less pronounced compared to our other studies^34,35^ possibly due to the relatively small study area, similar landscape type, and/or that all plants were of the same variety; all leading to bacterial community dissimilarity to be less pronounced. However, these results overall continue to build the pattern that intensifying production of coffee across the tropics can lead to alterations of farm environmental properties, alongside impacts to local biodiversity, including influencing the plant-associated microbiome which holds key benefits to plant productivity.

## Conclusion

Arabica coffee is a critical economic agroecological global crop. To ensure farmer livelihoods in the future, it is vital that we further develop understanding of the factors that influence coffee productivity, health, and quality^1–4^. In this study we show a relationship between coffee management system, the presence of distinct flavor profile, and the soil and cherry bacterial community. This study also establishes a potential relationship between the bacterial community and the flavor of coffee. Overall, what drives improved coffee flavor is relatively unknown, with likely many complex factors being involved. However, evidence from our study suggests that the bacterial community in the soil and cherries may play a role in coffee quality outcomes.

## Electronic supplementary material

Below is the link to the electronic supplementary material.

Supplementary Material 1

## Data Availability

Illumina sequence data has been deposited in the National Center for Biotechnology Information Sequence Read Archive under BioProject ID: PRJNA1200072.
