# Supplementary material for: Soil and cherry bacterial communities predict flavor on coffee farms
Source: Sci Rep. 2025 Jun 3;15:19387. doi: 10.1038/s41598-025-03665-6 (PMC12134262; doi:10.1038/s41598-025-03665-6)

**Supplemental Figure 1.** *The number of* *A. bacterial ASVs and B. bacterial families shared between four coffee farm management and flavor subgroups from soils (left panels) and coffee cherries (right panels) collected across two harvest periods in an annual cycle.*

*
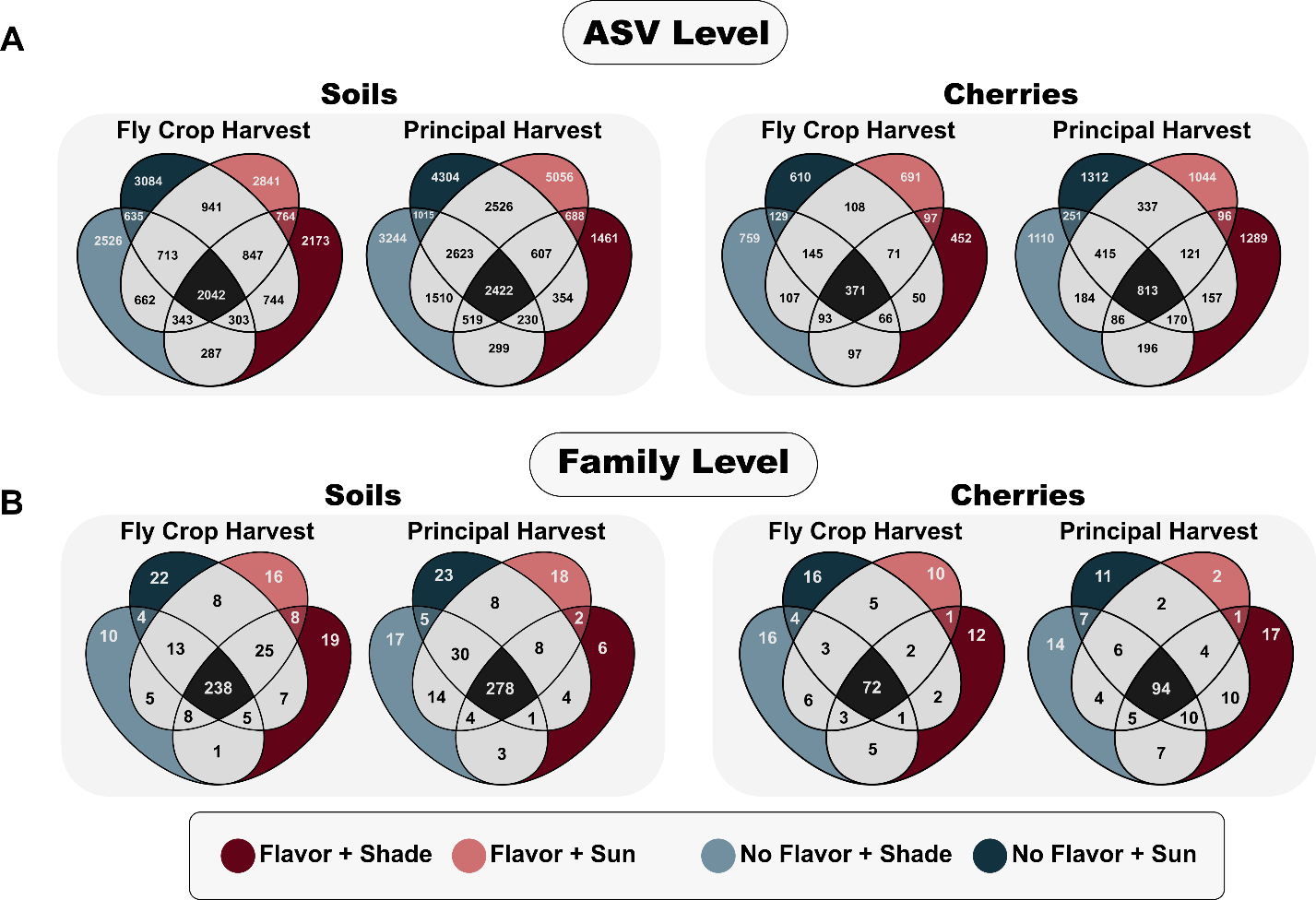
*

**Supplemental Figure 2:** *A: Beta diversity using Bray-Curtis distances of soil bacterial communities within each harvest period for four coffee flavor - farm system subgroups. B: Beta diversity Bray-Curtis distances of cherry bacterial communities within each harvest period.*

*
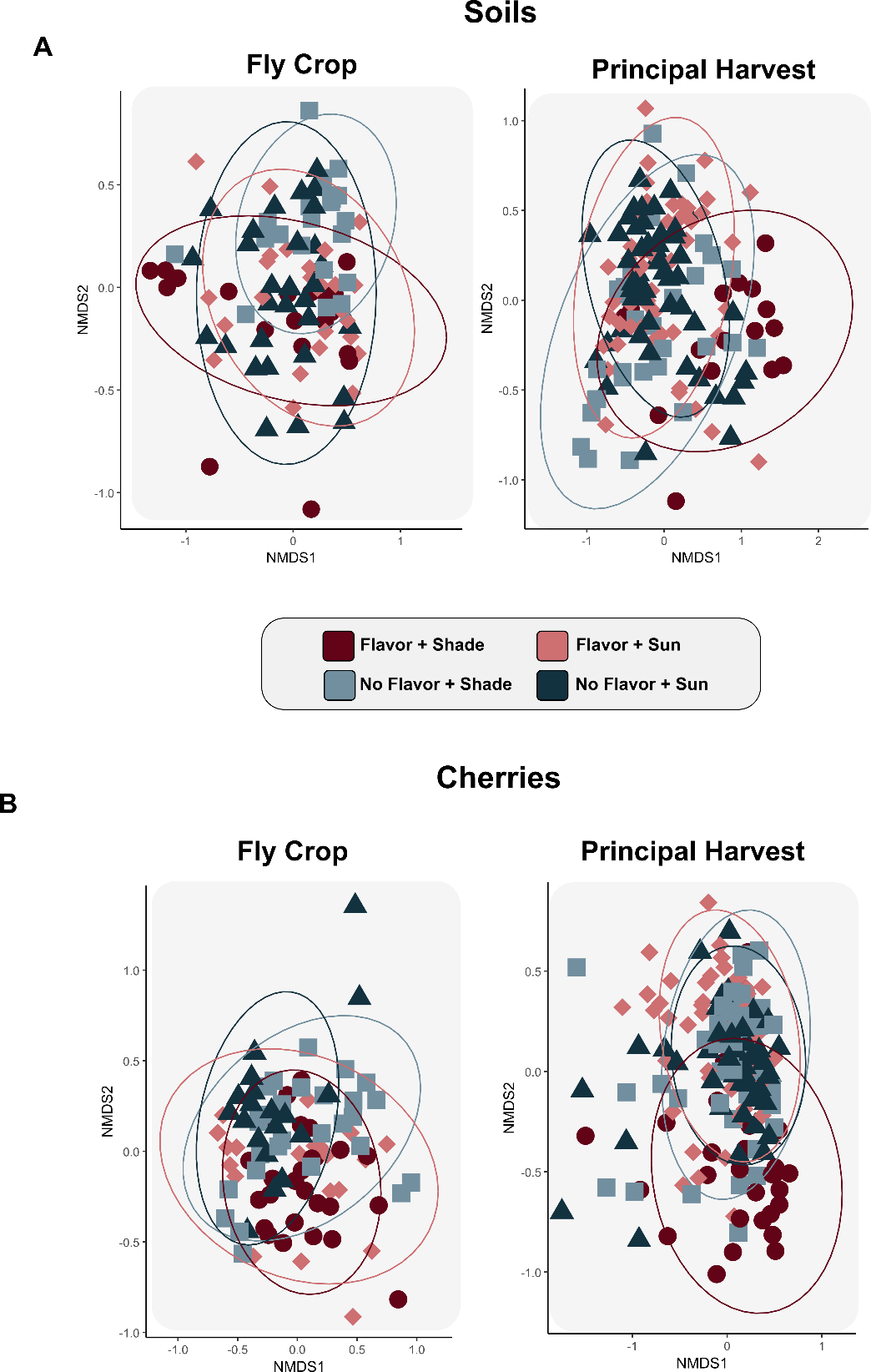
*

**Supplemental Figure 3:** *A: Differential abundance of soil genera to flavor presence within the principal harvest period shade grown coffee farms. B: Differential abundance of soil genera to flavor presence within the principal harvest period sun grown coffee farms. C: Differential abundance of cherry genera to flavor presence within the principal harvest period shade grown coffee farms. D: Differential abundance of cherry genera to flavor presence within the principal harvest period sun grown coffee farms.*


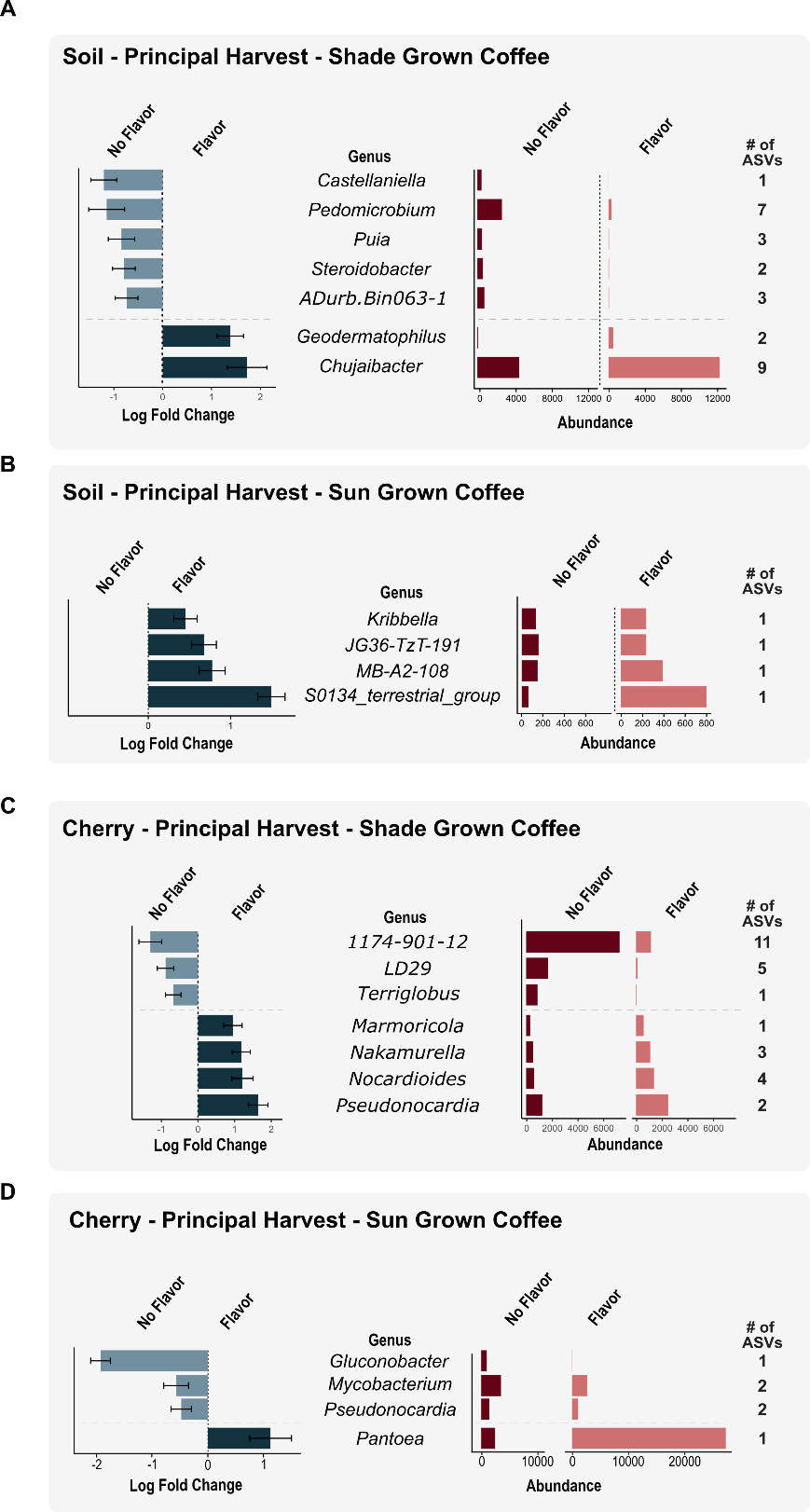

Supplement: Supplementary file 1 — Supplementary Material 1 [file 41598_2025_3665_MOESM1_ESM.docx]
